# Urinary microbiome-based metagenomic signature for the noninvasive diagnosis of hepatocellular carcinoma

**DOI:** 10.1038/s41416-024-02582-1

**Published:** 2024-01-26

**Authors:** Eun Ju Cho, Boram Kim, Su Jong Yu, Suk Kyun Hong, YoungRok Choi, Nam-Joon Yi, Kwang-Woong Lee, Kyung-Suk Suh, Jung-Hwan Yoon, Taesung Park

**Affiliations:** 1 Department of Internal Medicine and Liver Research Institute, Seoul National University College of Medicine, Seoul, 03080 Korea; 2 Interdisciplinary Program in Bioinformatics, Seoul National University, Seoul, 08826 Korea; 3 Department of Surgery, Seoul National University College of Medicine, Seoul, 03080 Korea; 4 Department of Statistics, Seoul National University, Seoul, 08826 Korea

**Keywords:** Oncology, Diagnostic markers

## Abstract

**Background:**

Gut microbial dysbiosis is implicated in chronic liver disease and hepatocellular carcinoma (HCC), but the role of microbiomes from various body sites remains unexplored. We assessed disease-specific alterations in the urinary microbiome in HCC patients, investigating their potential as diagnostic biomarkers.

**Methods:**

We performed cross-sectional analyses of urine samples from 471 HCC patients and 397 healthy controls and validated the results in an independent cohort of 164 HCC patients and 164 healthy controls. Urinary microbiomes were analyzed by 16S rRNA gene sequencing. A microbial marker-based model distinguishing HCC from controls was built based on logistic regression, and its performance was tested.

**Results:**

Microbial diversity was significantly reduced in the HCC patients compared with the controls. There were significant differences in the abundances of various bacteria correlated with HCC, thus defining a urinary microbiome-derived signature of HCC. We developed nine HCC-associated genera-based models with robust diagnostic accuracy (area under the curve [AUC], 0.89; balanced accuracy, 81.2%). In the validation, this model detected HCC with an AUC of 0.94 and an accuracy of 88.4%.

**Conclusions:**

The urinary microbiome might be a potential biomarker for the detection of HCC. Further clinical testing and validation of these results are needed in prospective studies.

## Background

Hepatocellular carcinoma (HCC) is a global health problem with increasing incidence and cancer-related mortality [1]. Recent advances in the management of HCC, suboptimal adherence to surveillance programs, late diagnosis, and a high recurrence rate even after curative treatment result in poor prognosis of patients with HCC. Enhancing early-stage diagnosis and improving treatment outcomes are important in the management of HCC.

Accumulating evidence indicates that the microbiota plays an important role in the progression of chronic liver disease and the development of HCC [2]. Gut dysbiosis and enhanced bacterial translocation into the systemic circulation increase hepatic exposure to microbial metabolites, leading to chronic inflammation, fibrosis and eventually HCC occurrence [3, 4]. In addition to the gut microbiota, changes in circulating microbiota are known to be associated with hepatic fibrosis in obese subjects, alcoholic hepatitis, and HCC [5–7]. Given the role of the urinary system in filtration of circulating microbiota and its metabolites, the urinary microbiota might reflect dysbiosis of the gut and circulating system as well [8]. It is known that the urine microbiota of breast cancer patients is significantly different from that of controls and is characterized by increased levels of gram-positive bacteria [9]. In addition, a recent study reported that the microbial compositions of stool, serum, and urine in a gastric cancer group were significantly different from those of a control group, and the urine microbiome-based model showed the best performance in the detection of gastric cancer when compared to the serum microbiome-based model [10]. However, the association of urine microbiota and HCC has not yet been studied.

In this context, the goal of the current study is to evaluate whether disease-specific alterations are present in the urine microbiome from patients with HCC and their potential as diagnostic biomarkers. A total of 1224 participants from four medical centers in South Korea were enrolled, and a three-step analysis, including the selection of HCC-associated markers, model building, and validation, was performed. Our model based on nine metagenome markers detected HCC with robust diagnostic accuracy, suggesting its potential as a noninvasive diagnostic tool for HCC.

## Methods

### Study population and sample collection

The model development (MD) set consisted of 471 patients newly diagnosed with HCC at the Seoul National University Hospital (Seoul, Korea) between February 2016 and December 2019 and 397 healthy controls who underwent a health checkup at the Inje University Haeundae Paik Hospital (Pusan, Korea) during the same period. The test set comprised 164 HCC patients from the Chungbuk National University Hospital (Cheongju, Korea) and the Chungnam National University Hospital (Daejeon, Korea) between January 2011 and December 2020 and 164 healthy controls and 28 subjects with chronic liver disease (at-risk group) from the Korea Association REsource (KARE) cohort [11, 12] from January 2015 to December 2016.

HCC was diagnosed according to international guidelines [13, 14]. Subjects with a history of chronic viral hepatitis or cirrhosis were defined as the at-risk group. The healthy group comprised subjects who had no known liver or kidney diseases and normal laboratory results. Midstream urine specimens were collected from participants before any treatment using standard protocols and stored at −80 °C until processing [15].

### Cell-free DNA extraction from urine samples

Urine samples were centrifuged at 10,000 × *g* for 10 min at 4 °C, and the supernatant was passed through a 0.22 μm membrane filter to remove foreign particles. Then, the samples were boiled at 100 °C for 40 min, and the supernatant was collected by centrifugation at 13,000 rpm for 30 min. DNA was extracted using a DNA extraction kit (PowerSoil DNA Isolation Kit, MO BIO, USA) and quantified by using the QIAxpert system (QIAGEN, Germany) following the manufacturer’s instructions.

### Metagenomic sequencing and data processing

The primers targeting the V3-V4 hypervariable regions of 16S rDNA (16S_V3_F 5′-TCGTCGGCAGCGTCAGATGTGTATAAGAGACAGCCTACGGGNGGCWGCAG-3′ and 16S_V4_R 5′-GTCTCGTGGGCTCGGAGATGTGTATAAGAGACAGGACTACHVGGGTATCTAATCC-3′) were used to amplify the bacterial genomic DNA. The libraries were constructed using the polymerase chain reaction products according to the MiSeq System guide (Illumina, USA), quantified using a QIAxpert (QIAGEN, Germany), and then sequencing was performed on a MiSeq (Illumina, USA) according to the manufacturer’s recommendations.

Demultiplexed paired-end reads obtained from the sequencer were processed using the QIIME2-2021.2 pipeline [16] with the following steps: (a) the removal of adapter and primer sequences using the Cutadapt plugin [17]; (b) quality control including denoising, dereplication, chimera removal with the DADA2 plugin [18], and merging of paired-end reads; (c) taxonomy assignment using the sklearn plugin [19] against the Silva database v.138.1 [20]; and (d) collapsing through the sum of the frequencies of the reads that have the same taxonomic assignment at the genus level. The final table at the genus level was used for downstream analysis.

Quality control was performed on a total of 786 genera identified in 868 samples of the MD set. To focus on the markers that were both prevalent and abundant, we used two criteria. First, the markers with a number of sequences exceeding 0.005% of the total sequences of all genera were retained. Second, markers with a zero proportion higher than 95% were excluded. We applied these filtering criteria to the MD set, leaving 121 genera.

To check the batch effect between the MD and test sets, nonmetric multidimensional scaling (NMDS) analyses using Bray‒Curtis distance were performed with the metaMDS function [21]. ComBat-Seq was used for batch effect adjustment [22]. For normalization, the datasets were transformed using the centered log ratio (CLR) transformation. To avoid the geometric mean becoming zero during log transformation, we used pseudo count 1. Unless otherwise specified, the rest of the statistical analysis was performed in R (version 4.1.0).

### Statistical analysis

To select a set of markers that were differentially abundant between HCC and control, single marker selection and multiple marker selection were performed. As single marker selection approaches, eight popular statistical methods were considered: methods developed for microbiome data, such as MetagenomeSeq [23], ANCOM [24], Metastats [25], CLR Perm [26] and ZIBSeq [27], and methods designed for bulk RNA-seq data, such as DESeq2 [28], edgeR [29], and the Wilcoxon rank sum test. *P* values were adjusted for multiple testing by the Benjamini‒Hochberg method [30]. Markers with *q* values < 0.05 in all eight methods were selected as candidates because the list of significant markers found from each method is quite different, and we can expect that the markers that were significant in many methods are reliable. Then, we examined the performance of the logistic regression model for all possible combinations of the selected markers. The model with the highest mean validation area under the curve (AUC) obtained through fivefold cross-validation (CV) was selected as the model with the best combination of markers. The AUC was computed using the pROC R package [31]. For multiple marker selection approaches, we used the least absolute shrinkage and selection operator (LASSO) and forward stepwise selection based on logistic regression. For LASSO, we found the set of markers by fitting the LASSO with optimal lambda based on the validation AUC obtained from fivefold CV. To fit the LASSO, the glmnet package was used [32]. In forward stepwise analysis, if the validation AUC obtained from fivefold CV did not increase by 0.01 or more when the marker was added to each step, we set that point as the optimal set of markers [33].

After marker selection, we built the prediction model with the selected set of markers based on logistic regression and evaluated the performance of the model with the test AUC. Sensitivity and specificity were determined with each model’s maximum balanced accuracy. Marker selection and model building were performed using the MD set, and age and sex were used as covariates. In the analysis of basic characteristics, the Wilcoxon rank-sum test or Kruskal‒Wallis test was used to compare continuous variables. Fisher’s exact test was used for categorical variables. Alpha diversity was presented as the Shannon index and the Simpson index, and the Wilcoxon rank sum test was used for comparison between the two groups. NMDS and Bray‒Curtis distance were used for beta diversity. Permutational multivariate analysis of variance (PERMANOVA) was used to test the difference in beta diversity between the three groups. All diversity work was conducted by the R package vegan [21].

## Results

### Baseline characteristics and outcomes

The baseline characteristics of subjects in the MD and test sets are presented in Table 1. There was no significant difference in sex or age between the HCC group and control group in either set.

**Table 1:** Baseline characteristics of the model development and test sets.

Clinical characteristics	Model development set			Test set			
	HCC (*n* = 471)	Healthy controls (*n* = 397)	*P*	HCC (*n* = 164)	At-risk group (*n* = 28)	Healthy controls (*n* = 164)	*P*
Age (years)	62 (19–88)	62 (33–85)	0.94	61 (35–86)	58 (53–69)	60 (53–79)	0.37
Male, No. (%)	365 (77.5)	304 (76.6)	0.75	123 (75.0)	24 (85.71)	123 (75.0)	0.49
Etiology, No. (%)							
HBV	356 (75.58)			88 (53.66)			
HCV	30 (6.37)			9 (5.49)			
HBV and HCV coinfection	6 (1.27)			3 (1.83)			
Alcohol	36 (7.64)			16 (9.76)			
NASH	13 (2.76)			1 (0.61)			
Others	30 (6.37)			47 (28.66)			
Child‒Pugh class, No. (%)							
A/B/C	445 (94.68)/25 (5.32)/0 (0)			68 (73.91)/21 (22.83)/3 (3.26)			
Creatinine (mg/dL)	0.86 (0.42–5.11)			0.84 (0.30–6.89)	1.04 (0.63–1.62)	1.00 (0.64–2.33)	4.07 × 10^–8^
MDRD GFR	87.83 (11.21–186.97)			92.78 (7.85–218.68)	72.42 (42.46–126.27)	72.29 (29.23–96.34)	1.94 × 10^–10^
BCLC stage, No. (%)							
0-A/B/C/D	94 (19.96)/247 (52.44)/57 (12.10)/73 (15.50)			85 (71.43)/5 (4.20)/29 (24.37)/0			
Not available	-			45			
AFP (ng/mL)	11 (1–261,635)			6.94 (1.07–200,000)			
PIVKA-II (mAU/mL)	52 (1.01–75,000)			27 (11–12,758)			
Treatment, No. (%)							
Resection	282 (59.87)			136 (82.92)			
Liver transplantation	3 (0.63)			0			
RFA	27 (5.73)			2 (1.21)			
TACE	143 (30.36)			18 (10.97)			
Systemic chemotherapy	16 (3.39)			1 (0.60)			
Supportive care	0			7 (4.26)			

Data are presented as medians with minimum and maximum or numbers (%).

*AFP* alpha-fetoprotein, *BCLC* Barcelona clinic liver cancer, *HBV* hepatitis B virus, *HCC* hepatocellular carcinoma, *HCV* hepatitis C virus, *MDRD* *GFR* modification of diet in renal disease glomerular filtration rate, *NASH* non-alcoholic steatohepatitis, *PIVKA-II* protein induced by vitamin K absence or antagonist II, *RFA* radiofrequency ablation, *TACE* transarterial chemoembolization.

### Comparisons of microbial diversity between HCC patients and controls

**Fig. 1:**
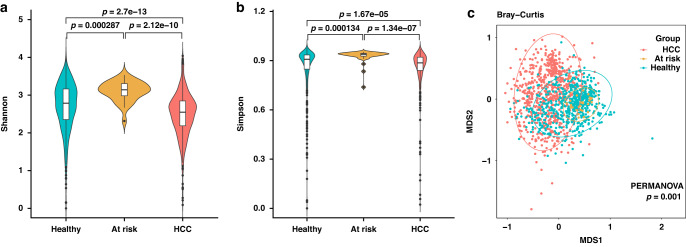
Bacterial diversity of the urine microbiota. **a** Alpha diversity estimated by the Shannon index and **b** Simpson index. **c** Beta diversity calculated by NMDS plot with the Bray‒Curtis distance. HCC hepatocellular carcinoma, PERMANOVA permutation multivariate analysis of variance.

To assess the differences in microbial diversity between groups, we evaluated the abundance of taxa. Alpha diversity, estimated by the Shannon index and Simpson index, was significantly decreased in the HCC group compared to the healthy control group (*p* = 2.70 × 10^−13^ and 1.67 × 10^−^^5^, respectively; shown in Fig. 1a, b). Similarly, there was a significant difference between the HCC and at-risk control groups (*p* = 2.12 × 10^−^^10^ and 1.34 × 10^−^^7^, respectively, shown in Fig. 1a, b). To display microbiome space between samples, beta diversity was assessed with an NMDS plot and the Bray‒Curtis distance at the genus level. The results showed a significantly different distribution among groups, which suggested the presence of genus-level markers distinguishing the HCC group from the other groups (*p* = 1.00 × 10^−^^3^; shown in Fig. 1c).

### Differentially abundant markers between HCC patients and healthy controls in the MD set

To identify the genus-level markers differentiating HCC from controls, we applied eight statistical methods and identified significant markers. Of the 121 tested genera, we identified 13 markers that were significant in all eight methods (Supplementary Table 1). Then, we searched exhaustively to select the optimal marker set based on fivefold CV [34]. The model comprised of 9 HCC-associated genera (i.e., Neisseria, Cutibacterium, Fusobacterium, Akkermansia, Bacteroides, Streptococcus, Prevotella_9, Capnocytophaga, and Bradyrhizobium) showed the highest validation AUC of 0.8869 in fivefold CV (Supplementary Table 2).

We additionally performed multiple marker selection approaches using LASSO and forward stepwise selection based on fivefold CV for all 121 genera. In the LASSO and forward stepwise analyses, 14 markers and 5 markers were selected, respectively (Supplementary Table 3). Finally, we trained a logistic regression model consisting of a set of markers selected from each of the three approaches, single marker selection, LASSO, and forward stepwise selection, using the entire MD set.

### Performance of models in the test set

**Fig. 2:**
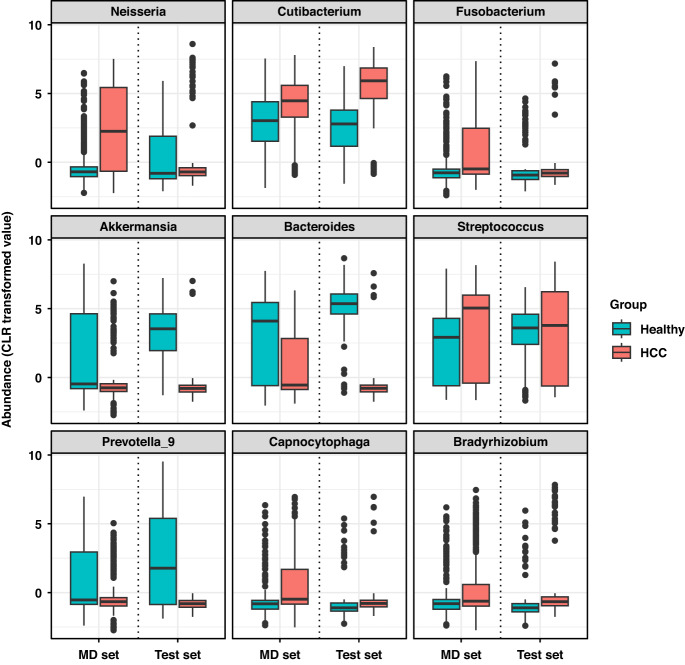
Boxplots of compositional differences for genus-level markers. CLR centered log ratio, HCC hepatocellular carcinoma, MD model development.

Next, we evaluated the performance of the models in the test set consisting of 164 HCC patients and 164 healthy controls. The model based on single marker selection showed the highest performance (AUC = 0.9399, sensitivity = 0.8902, specificity = 0.878, and accuracy = 0.8841), suggesting the possibility for the urine microbiome-based signature to accurately distinguish HCC patients from healthy controls (Supplementary Table 3). All nine taxa (i.e., Neisseria, Cutibacterium, Fusobacterium, Akkermansia, Bacteroides, Streptococcus, Prevotella_9, Capnocytophaga, and Bradyrhizobium) included in this model showed similar increasing or decreasing trends in abundance between groups in both the MD and test sets (shown in Fig. 2). The models based on multiple marker selection using LASSO and forward stepwise selection also showed good performance (AUCs, 0.8415 and 0.7293, respectively) with the test set (Supplementary Table 3). In addition, to investigate whether the models could also distinguish HCC patients from patients with chronic liver disease, the performance of the models was assessed in 164 HCC patients and 28 subjects with chronic liver disease. The model based on single marker selection showed an AUC of 0.9386 in the detection of HCC patients versus at-risk subjects, which was comparable to the model’s performance in distinguishing HCC patients from healthy controls. The models based on LASSO and forward stepwise selection also showed high performances of 0.8406 and 0.7152, respectively.

## Discussion

This study evaluated the association between urine microbiota and HCC for the first time. HCC was associated with altered urinary microbial composition and a significantly lower level of diversity compared to controls. Furthermore, we showed the diagnostic performance of the urine microbiota-based signature to differentiate HCC and suggested its potential as a noninvasive diagnostic tool for HCC.

Urine contains several metabolites, proteins, and circulating DNA originating from the whole body that may have diagnostic or prognostic potential for various nonurological cancers [35]. Several metabolomic studies have shown that urine metabolite biomarkers can distinguish HCC patients from controls, although the lack of specificity related to global cancer-associated metabolic alterations limits their clinical application [35]. Another study reported that seven urinary protein signatures yield good performance in discriminating HCC from chronic hepatitis B patients [36]. Furthermore, urine circulating tumor DNA markers (i.e., mutated TP53, methylated RASSF1a, and GSTP1) were able to detect early-stage HCC patients, especially those with low AFP levels [37]. These findings suggest the potential of urine biomarkers to facilitate HCC surveillance and diagnosis via noninvasive methods.

Various studies have reported that the gut microbiome can affect the development and progression of HCC. The gut microbiome can activate the lipopolysaccharide-toll-like receptor four pathway and promote HCC growth in mice [4]. In addition, gut microbiome-mediated bile acid metabolism affects anticancer immunity via hepatic natural killer T cells [38]. Moreover, recent studies have suggested the gut or circulating microbiome as diagnostic biomarkers for early-stage HCC [7, 39]. These results suggest that the microbiota in the gut and blood present moderate dysbiosis in HCC patients, and these signatures might be biomarkers for detecting HCC. Considering urine as an ultrafiltrate of blood, the urinary microbiota might reflect dysbiosis of the gut and circulatory system as well. Therefore, we tried to find a urine microbiome-derived metagenomic signature, as urine has several advantages over blood or feces: it can be easily and repeatedly obtained via a noninvasive method and provides a stable matrix for analysis.

Consistent with previous studies assessing gut and circulating microbiota associated with HCC [7, 40], the abundance of Akkermansia, a well-known gut commensal that promotes intestinal integrity and attenuates hepatic injury [41], was markedly decreased in the HCC group. In contrast, Bacterioide was increased in the HCC group. Bacteroides has a proinflammatory effect in subjects eating a Western-type diet, promoting the progression of nonalcoholic fatty liver disease [42]. Furthermore, increased Bacteroides abundance has been associated with the upregulation of inflammatory cytokines, activated monocytes, and monocytic myeloid-derived suppressor cells, suggesting a potential role in hepatocarcinogenesis [40]. In this study, the abundance of potentially pathogenic bacteria known to be increased in the gut of HCC patients, such as Streptococcus [40], Fusobacterium [43], and Prevotella [44], was also increased in the HCC group. These findings suggest that urine microbiota might capture gut dysbiosis, i.e., the decrease in potentially beneficial bacteria protecting intestinal integrity and the increase in potentially harmful bacteria might promote intestinal and hepatic inflammation and the development of HCC.

However, there are several limitations in our study. First, this study could not elucidate whether urine dysbiosis is a co-phenomenon or a true player in the development and progression of HCC. Future mechanistic studies are required to evaluate the functional potential of the urine microbiota. Second, as this study could not analyze samples from patients with other cancer types, and only a small number of samples from patients with chronic liver disease were available, our results may not fully discriminate HCC from other cancer types or high-risk subjects. Additionally, serum alpha-fetoprotein (AFP) levels were not available for the healthy controls; therefore, we could not compare the performance of AFP levels with that of our models. Further studies with a larger number of cases covering various cancers and at-risk populations are necessary for more definitive results. Last, we could not analyze the microbiome in other organ systems. Although the urine microbiome is probably mostly derived from the gut and/or circulating microbiome, there may be a significant difference between the circulating microbiome potentially involved in host immune-microbial interactions and those existing in the gut. Further mechanistic studies regarding the crosstalk between the gut-liver axis and urine microbiome and their functional roles in HCC are needed.

In conclusion, this study showed compositional dysbiosis in the urine microbiome of patients with HCC and its diagnostic potential for HCC. Further functional studies and validation in larger, independent cohorts are required to validate the role of the urine microbiota-based metagenomic signature in HCC.

### Supplementary information

Supplementary Table

## Data Availability

The datasets generated during and/or analyzed during the current study are available in the NCBI Sequence Read Archive under project numbers PRJNA1048836 and PRJNA716550.
