# Supplementary material for: Urinary microbiome-based metagenomic signature for the noninvasive diagnosis of hepatocellular carcinoma
Source: Br J Cancer. 2024 Jan 26;130(6):970–5. doi: 10.1038/s41416-024-02582-1 (PMC10951239; doi:10.1038/s41416-024-02582-1)
Supplement: Supplementary file 1 — Supplementary Table [file 41416_2024_2582_MOESM1_ESM.docx]

**Supplementary Table 1.** P values of significant markers from single marker selection.

| **ID** | **Genus** | **CLR Perm** | **DESeq2** | **edgeR** | **Metastats** | **Wilcoxon** | **ZIBSeq** | **ZIG** | **ANCOM** |
| --- | --- | --- | --- | --- | --- | --- | --- | --- | --- |
| Genus_106 | Neisseria | 0 | 2.01×10^-61^ | 1.23×10^-13^ | 2.81×10^-3^ | 4.61×10^-28^ | 3.57×10^-8^ | 1.07×10^-95^ | 0 |
| Genus_14 | Cutibacterium | 0 | 3.61×10^-20^ | 1.64×10^-2^ | 2.81×10^-3^ | 2.64×10^-19^ | 3.05×10^-14^ | 2.59×10^-21^ | 0 |
| Genus_85 | Fusobacterium | 0 | 4.73×10^-32^ | 2.71×10^-3^ | 2.81×10^-3^ | 3.23×10^-11^ | 1.54×10^-4^ | 5.39×10^-58^ | 0 |
| Genus_120 | Akkermansia | 0 | 6.7×10^-57^ | 8.63×10^-17^ | 2.81×10^-3^ | 4.39×10^-33^ | 7.74×10^-16^ | 9.02×10^-129^ | 0 |
| Genus_18 | Bacteroides | 0 | 2.71×10^-43^ | 1.16×10^-23^ | 2.81×10^-3^ | 5.98×10^-33^ | 4.18×10^-30^ | 3.42×10^-35^ | 0 |
| Genus_49 | Streptococcus | 0 | 1.18×10^-15^ | 2.71×10^-2^ | 2.81×10^-3^ | 1.83×10^-16^ | 3.03×10^-20^ | 5.28×10^-58^ | 0 |
| Genus_23 | Prevotella_9 | 0 | 1.56×10^-64^ | 2.08×10^-16^ | 2.81×10^-3^ | 5.3×10^-16^ | 3.93×10^-20^ | 1.52×10^-79^ | 0 |
| Genus_26 | Capnocytophaga | 0 | 7.52×10^-17^ | 4.74×10^-2^ | 2.81×10^-3^ | 1.29×10^-16^ | 4.21×10^-2^ | 2.14×10^-39^ | 0 |
| Genus_92 | Bradyrhizobium | 0 | 3.92×10^-34^ | 8.44×10^-3^ | 2.81×10^-3^ | 3.61×10^-6^ | 2.78×10^-5^ | 6.43×10^-60^ | 0 |
| Genus_1 | Actinomyces | 0 | 6.62×10^-21^ | 2.97×10^-2^ | 2.81×10^-3^ | 2.29×10^-7^ | 6.59×10^-8^ | 1.52×10^-40^ | 0 |
| Genus_12 | Rothia | 0 | 4.09×10^-27^ | 3.11×10^-3^ | 2.81×10^-3^ | 4.05×10^-13^ | 3.17×10^-7^ | 4.83×10^-68^ | 0 |
| Genus_74 | Faecalibacterium | 0 | 3.55×10^-5^ | 1.43×10^-4^ | 2.81×10^-3^ | 1.34×10^-8^ | 1.78×10^-2^ | 5.09×10^-4^ | 0 |
| Genus_50 | Gemella | 0 | 4.75×10^-35^ | 1.64×10^-2^ | 2.81×10^-3^ | 1.82×10^-11^ | 1.16×10^-3^ | 1.1×10^-65^ | 0 |

**Supplementary Table 2.** The performance of the best model for each number of markers from exhaustive search.

| **Number of microbiome markers** | **Model** | **Train (5-fold CV)** | **Validation (5-fold CV)** |
| --- | --- | --- | --- |
| Covariate model | Age + Gender | 0.5127 | 0.4680 |
| 1 | Age + Gender + Genus_106 | 0.7444 | 0.7369 |
| 2 | Age + Gender + Genus_106 + Genus_14 | 0.8316 | 0.8251 |
| 3 | Age + Gender + Genus_106 + Genus_14 + Genus_85 | 0.8539 | 0.8495 |
| 4 | Age + Gender + Genus_106 + Genus_14 + Genus_85 + Genus_120 | 0.8718 | 0.8652 |
| 5 | Age + Gender + Genus_106 + Genus_14 + Genus_85 + Genus_120 + Genus_18 | 0.8781 | 0.8712 |
| 6 | Age + Gender + Genus_106 + Genus_14 + Genus_85 + Genus_120 + Genus_18 + Genus_49 | 0.885 | 0.8783 |
| 7 | Age + Gender + Genus_106 + Genus_14 + Genus_85 + Genus_120 + Genus_18 + Genus_49 + Genus_23 | 0.8886 | 0.8824 |
| 8 | Age + Gender + Genus_106 + Genus_14 + Genus_85 + Genus_120 + Genus_18 + Genus_49 + Genus_23 + Genus_26 | 0.8915 | 0.8847 |
| 9* | Age + Gender + Genus_106 + Genus_14 + Genus_85 + Genus_120 + Genus_18 + Genus_49 + Genus_23 + Genus_26 + Genus_92 | 0.8955 | 0.8869 |
| 10 | Age + Gender + Genus_106 + Genus_14 + Genus_85 + Genus_120 + Genus_18 + Genus_49 + Genus_23 + Genus_26 + Genus_92 + Genus_1 | 0.8966 | 0.8868 |
| 11 | Age + Gender + Genus_106 + Genus_14 + Genus_85 + Genus_120 + Genus_18 + Genus_49 + Genus_23 + Genus_26 + Genus_92 + Genus_1 + Genus_12 | 0.8967 | 0.8866 |
| 12 | Age + Gender + Genus_106 + Genus_14 + Genus_85 + Genus_120 + Genus_18 + Genus_49 + Genus_23 + Genus_26 + Genus_92 + Genus_1 + Genus_12 + Genus_74 | 0.8968 | 0.8855 |
| 13 | Age + Gender + Genus_106 + Genus_14 + Genus_85 + Genus_120 + Genus_18 + Genus_49 + Genus_23 + Genus_26 + Genus_92 + Genus_1 + Genus_12 + Genus_74 + Genus_50 | 0.8969 | 0.8847 |

*The finally selected model showing the highest validation AUC.

**The symbols are as following: genus_106, Neisseria; genus_14, Cutibacterium; genus_85, Fusobacterium; genus_120, Akkermansia; genus_18, Bacteroides; genus_49, Streptococcus; genus_23, Prevotella_9; genus_26, Capnocytophaga; genus_92, Bradyrhizobium; genus_1, Actinomyces; genus_12, Rothia; genus_74, Faecalibacterium; genus_50, Gemella.

**Supplementary Table 3.** Performance of the best model to distinguish HCC patients from healthy controls obtained by each method.

| **Model** | | | **Model development set** | | | | | | | **Test set** | | | | |
| --- | --- | --- | --- | --- | --- | --- | --- | --- | --- | --- | --- | --- | --- | --- |
|  |  |  | **AUC** | **AUC_Tr_ (5-fold CV)** | **AUC_Val_ (5-fold CV)** | **Sensitivity** | **Specificity** | **Accuracy** | **AUC** | | **Sensitivity** | **Specificity** | **Accuracy** |  |
| **Covariates** | | Age + Gender | 0.5197 | 0.5127 | 0.4680 | 0.5648 | 0.5038 | 0.5343 | 0.4869 | | 0.8841 | 0.1707 | 0.5274 |  |
| **Single marker selection*** | **Univariate test & Exhaustive search** | Age + Gender + Genus_106 + Genus_14 + Genus_85 + Genus_120 + Genus_18 + Genus_49 + Genus_23 + Genus_26 + Genus_92 | 0.8944 | 0.8955 | 0.8869 | 0.8386 | 0.7859 | 0.8123 | 0.9399 | | 0.8902 | 0.878 | 0.8841 |  |
| **Multiple marker selection** | **Forward stepwise** | Age + Gender + Genus_31 + Genus_14 + Genus_41 + Genus_111 + Genus_85 | 0.8966 | 0.8972 | 0.8932 | 0.7941 | 0.8564 | 0.8252 | 0.7293 | | 0.75 | 0.7256 | 0.7378 |  |
|  | **LASSO** | Age + Gender + Genus_14 + Genus_18 + Genus_19 + Genus_31 + Genus_53 + Genus_62 + Genus_85 + Genus_86 + Genus_96 + Genus_106 + Genus_111 + Genus_116 + Genus_117 + Genus_120 | 0.937 | 0.9382 | 0.9274 | 0.9172 | 0.8086 | 0.8629 | 0.8415 | | 0.7439 | 0.8232 | 0.7835 |  |

Abbreviations: AUC, area under the curve; LASSO, least absolute shrinkage and selection operator; Tr, training; Val, validation; CV, cross validation.

*The finally selected model.

**The symbols are as following: genus_106, Neisseria; genus_14, Cutibacterium; genus_85, Fusobacterium; genus_120, Akkermansia; genus_18, Bacteroides; genus_49, Streptococcus; genus_23, Prevotella_9; genus_26, Capnocytophaga; genus_92, Bradyrhizobium; genus_31, Helicobacter; genus_41, Atopostipes; genus_111, Aggregatibacter; genus_19, Porphyromonas; genus_53, Clostridium_sensu_stricto_1; genus_62, Lachnospiraceae_UCG-001; genus_86, Leptotrichia; genus_96, Burkholderia-Caballeronia-Paraburkholderia; genus_116, Pseudomonas; genus_117, Stenotrophomonas.
